# Application of Endoscopic Submucosal Dissection for Removal of Deep Invasive Submucosal Colon Carcinoma

**DOI:** 10.1155/2009/573981

**Published:** 2009-08-19

**Authors:** Sergio A. Con, Yutaka Saito, Takahisa Matsuda, Hirokazu Taniguchi, Takeshi Nakajima

**Affiliations:** ^1^Endoscopy Division, National Cancer Center Hospital, 5-1-1 Tsukiji, Chuo-ku, 104-0045 Tokyo, Japan; ^2^Surgery and Endoscopy Divisions, Centro Digestivo Doctores Con-Mediplaza, San Jose, Costa Rica; ^3^Clinical Laboratory Division, National Cancer Center Hospital, 5-1-1 Tsukiji, Chuo-ku, 104-0045 Tokyo, Japan

## Abstract

Endoscopic submucosal (sm) dissection (ESD) is a recently used technique that enables *en-bloc* resection of large colorectal tumors allowing a more precise histopathological analysis of the resected specimen. However, it has not been widely adopted even in Japan mainly due to its technical difficulty and increased risk of perforation. Herein, we present an ESD-treated lesion with deep sm invasion removed without complications, such as bleeding or perforation, from a patient at high-risk for surgical intervention. A successful ESD was achieved although the sm invasion was greater than 1000 *μ*m from the muscularis mucosae, and the nonlifting sign was positive. It is our belief that this procedure should be performed at least in patients at high-risk for surgical intervention. At present, we have removed 16 lesions with deep sm invasion by ESD without complications, demonstrating that deep sm cancer can be successfully resected by this technique as a local resection. Herein, we report on one of these cases.

## 1. Introduction

In Japan, as shown in [1–3], EMR is the treatment of choice for superficial early colorectal cancer due to the minimal invasiveness and efficacy of the procedure. However, when dealing with flat lesions greater than 20 mm in diameter, the rate of piecemeal resection, incomplete removal, and local recurrence after EMR increases, as discussed in [4–6]. Endoscopic submucosal (sm) dissection (ESD) is a recently used technique that enables *en-bloc* resection of large colorectal tumors allowing a more precise histopathological analysis of the resected specimen, as shown in [7, 8]. However, as cited in [9, 10], colorectal ESD has not been widely adopted even in Japan mainly due to its technical difficulty and increased risk of perforation. 

 Herein, we report on an ESD-treated lesion with deep sm invasion removed from a patient at highrisk for surgical intervention. The role of ESD for lesions with sm invasion greater than 1000 *μ*m from the muscularis mucosae is discussed. 

## 2. Case Presentation

**Figure 1:**
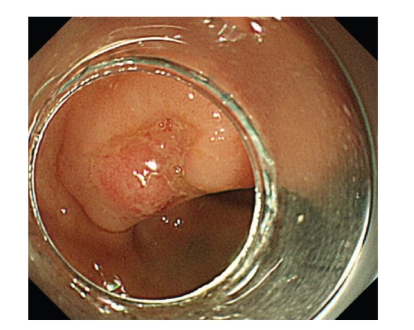
Conventional view of a 0-IIa + IIc lesion located in the rectosigmoid colon. A definite depressed margin and irregular reddened surface of depression suggested submucosal deep invasion.

 A 79-year-old male was diagnosed to have a lesion of 28 mm in diameter located in the rectosigmoid colon, with a 0-IIa + IIc macroscopic type (Figure 1). Pathological findings of biopsy specimens revealed a well-differentiated adenocarcinoma. The nonlifting sign was positive. As discussed by Matsuda [11], high magnifying observation with crystal violet staining showed an invasive pattern with irregular and distorted epithelial crypts in the demarcated area suggesting sm invasion greater than 1000 *μ*m from the muscularis mucosae. Open surgery was the first choice offered to the patient. However, as he was considered to be at high risk for surgical intervention due to his history of two acute myocardial infarction episodes and an abdominal aorta bypass procedure, endoscopic resection of the lesion was recommended to avoid open surgery.

**Figure 2:**
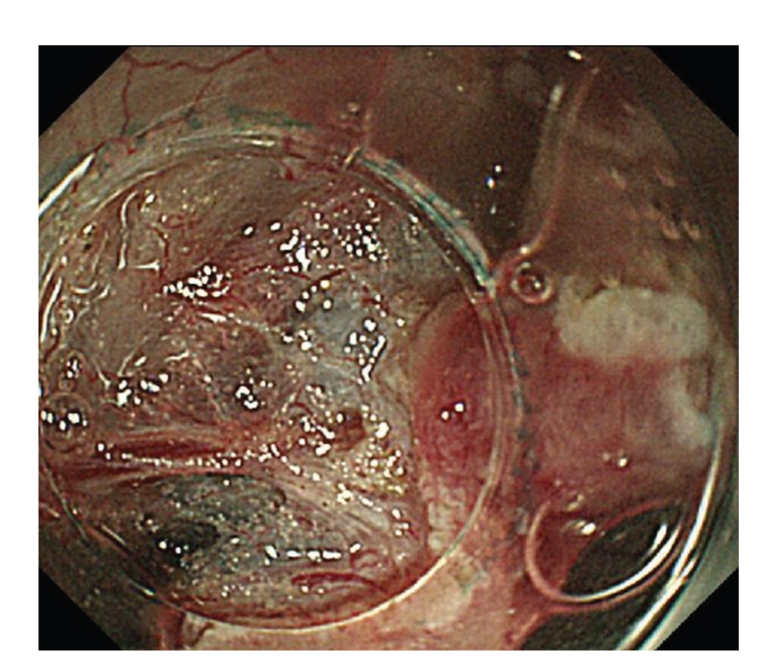
Direct observation of the submucosal and muscle layers is possible due to the utility of distal attachment.

**Figure 3:**
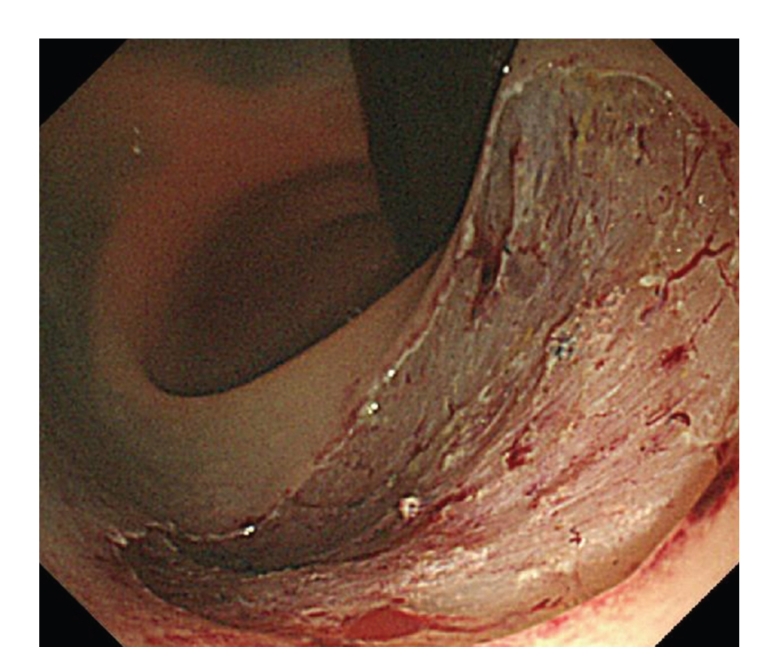
Ulcer bed after *en-bloc* resection of the lesion. There is no muscle damage or bleeding.

**Figure 4:**
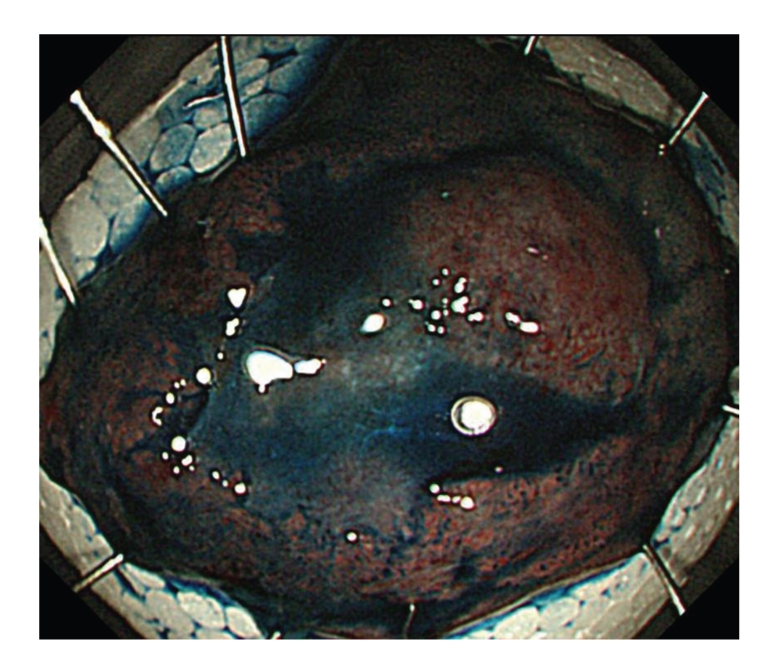
A view on the *en-bloc* resected specimen. The surgical margin is visible.

**Figure 5:**
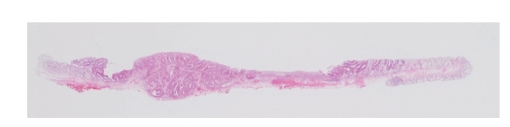
Histopathological analysis of the resected specimen showed a well-differentiated adenocarcinoma, and the depth of invasion was sm 2500 *μ*m with negative lateral and vertical margins.

 As decribed in [12–15], under conscious sedation, with CO_2_ insufflation, after indigocarmine dye spraying and injection of glycerol and sodium hyaluronate acid into the sm layer, a circumferential mucosal incision was made using a bipolar current needle knife (B-knife) (XEMEX Co., Tokyo, Japan). The sm dissection was performed using a B-knife and an insulation-tipped diathermic knife (IT-knife) (Olympus Medical Systems Corp., Tokyo, Japan) providing a direct observation of the sm and muscle layers (Figure 2). The resection left an ulcer bed without muscle damage or bleeding (Figure 3). The *en-bloc* resection and removal of the specimen was completed in approximately 80 minutes without any complication (Figure 4). Histopathological analysis of the resected specimen showed a well-differentiated adenocarcinoma, and the depth of invasion was sm 2500 *μ*m with negative lateral and vertical margins (Figure 5). 

## 3. Discussion

According to the Paris classification of superficial neoplastic lesions [16], lesions with sm invasion of less than 1000 *μ*m from the muscularis mucosae should be removed by ESD. In contrast, lesions with sm invasion exceeding 1000 *μ*m are currently considered for surgical resection, as shown in [17, 18]. In this particular case, due to the patient's critical heart condition, ESD was performed although the sm invasion exceeded 1000 *μ*m in depth, and the nonlifting sign was positive. 

 At the National Cancer Center Hospital a total of 16 cases with deep invasive sm lesions have been successfully removed by ESD, with negative lateral and vertical margins observed during histopathological analysis of the resected specimen. 

During ESD for sm cancer, a direct observation of the sm and muscle layers possibilitates a total sm resection. If the lesion has invaded the muscularis propria though, ESD should not be performed because of the risk of colonic perforation. However, as discussed by Saito [7], the use of the B-knife in ESD has decreased the perforation rate. By using this device, a safer procedure is achieved probably because the electric current is centered to the tip of the needle, as shown in [19]. 

 This case has demonstrated that the removal of lesions with sm invasion greater than 1000 *μ*m from the muscularis mucosae can be successfully resected by ESD as a local resection. It is our belief that this procedure should become the treatment of choice for deep invasive sm cancer at least in patients at high risk for surgical intervention, which would avoid the risk of a surgical procedure and would improve the patient's quality of life.
